# Understanding Opportunities for Prescribing Pre-exposure Prophylaxis at Two Academic Medical Centers in a High Priority Jurisdiction for Ending the HIV Epidemic

**DOI:** 10.1007/s10461-025-04767-y

**Published:** 2025-06-10

**Authors:** Moira C. McNulty, Katherine McGuckin, Eleanor E. Friedman, Matthew Caputo, Joseph A. Mason, Samantha A. Devlin, Mihai Giurcanu, Aniruddha Hazra, Jessica P. Ridgway, Chad J. Achenbach

**Affiliations:** 1 Department of Medicine, Section of Infectious Diseases and Global Health, University of Chicago, 5841 S. Maryland Ave, MC 5065, Chicago, IL 60637 USA; 2 Havey Institute for Global Health, Feinberg School of Medicine, Northwestern University, Chicago, IL USA; 3 Department of Public Health Sciences, University of Chicago, Chicago, IL USA; 4 Department of Medicine, Division of Infectious Diseases, Feinberg School of Medicine, Northwestern University, Chicago, IL USA; 5 Present Address: Woebot Health, San Francisco, USA; 6 Present Address: University of Minnesota, Minneapolis, USA

**Keywords:** HIV, Pre-exposure prophylaxis, PrEP, Prescription, Disparities

## Abstract

Pre-exposure prophylaxis (PrEP) is an effective yet underutilized tool for HIV prevention. We examined opportunities for prescribing PrEP at two large, urban, academic healthcare institutions. We analyzed electronic medical record data for 1/1/2015-12/31/2021 among patients *≥* 18 years of age, with *≥* 1 negative HIV test with indications for PrEP. Eligible encounters were six months after a sexually transmitted infection, or when injection drug use (IDU) was documented. We categorized encounter setting, including the emergency department (ED) and obstetrics and gynecology/women’s health (OBGYN) department. We performed logistic mixed effects regression, reporting odds ratios and confidence intervals (OR, aOR, 95% CI). Overall, 9644 people contributed 53,031 encounters with 4653 PrEP prescriptions. The two institutions had different patient population demographics, with institution A having a higher proportion of women, patients aged 18–24, and non-Hispanic Blacks (NHB), and institution B having a higher proportion of men who have sex with men (MSM), non-Hispanic Whites (NHW), and Hispanic/Latinos. Adjusted models found lower odds of PrEP prescriptions for NHB (aOR 0.21 [0.15, 0.29]), Hispanic/Latino (aOR 0.53 [0.37, 0.76]), heterosexual women (aOR 0.10 [0.06, 0.17]), IDU (aOR 0.01 [0.001, 0.08]), and encounters at the ED or OBGYN (0.14 [0.06, 0.31]). Increased odds of PrEP prescription were seen among NHW (aOR 4.85 [3.45, 6.82]), MSM (aOR 24.87 [15.79, 39.15]), and patients at institution B (aOR 1.78 [1.25, 2.53]). Institution A contained people historically underrepresented in PrEP prescriptions, while institution B accounted for most PrEP prescriptions. Opportunities exist to improve equity in PrEP prescriptions among demographic groups and in hospital settings.

**Supplementary Information:**

The online version contains supplementary material available at 10.1007/s10461-025-04767-y.

## Introduction

HIV pre-exposure prophylaxis (PrEP) is a key intervention for ending HIV transmission. The national Ending the HIV Epidemic (EHE) plan and many state and local plans call for increasing PrEP use among populations most impacted by HIV to prevent new infections [1–3]. An estimated 1.1 million persons are considered at risk for HIV in the U.S. and may benefit from PrEP [4]. PrEP is endorsed by the United States Preventive Services Task Force, and criteria for eligibility to indicate those who can most benefit from PrEP have been outlined by the Centers for Disease Control and Prevention (CDC) [5, 6]. Although PrEP use has increased over the past several years, uptake is still below that estimated to meet the EHE goal of reducing HIV transmission by 90% by 2030 [7–10]. Furthermore, PrEP uptake remains variable across groups and geographic areas, often with the most vulnerable to HIV having the lowest PrEP uptake [7, 11, 12].

Within healthcare settings, many provider-level barriers to prescribing PrEP have been reported, including limited knowledge or awareness of who may benefit from PrEP, lack of recognition of patient risk factors for HIV (i.e., sexual and social behaviors), and discomfort with prescribing PrEP [13–16]. Barriers to prescribing PrEP and persistent disparities in prescriptions for those most vulnerable to HIV have been documented; however, solutions for increasing appropriate PrEP prescribing at large, multispecialty academic medical centers have not been well described. Understanding healthcare system differences in PrEP prescribing and uptake may offer insight into possible interventions to improve PrEP equity.

The objectives of this study were to (1) understand PrEP prescribing patterns and (2) identify opportunities for PrEP initiation at two academic medical centers within the greater Chicago area, a high priority EHE jurisdiction.

## Methods

Data for this study included Electronic Data Warehouse data from the University of Chicago Medicine and Northwestern Medicine for adults aged 18 years or older who were tested for HIV between January 1st, 2015, and December 31st, 2021. Patients were eligible for inclusion in the cohort if they had at least one negative HIV test during the study period and were not diagnosed with HIV, to ensure that only people eligible for PrEP were examined, mirroring the first step of the PrEP cascade. Additional data included patient demographic information, social history, medications, laboratory results for sexually transmitted infections (STIs), ICD-10 codes for diagnosis of HIV, and encounter information.

Using the first negative HIV test during the study as the entry date for the cohort, we examined subsequent encounter information (e.g., diagnoses, labs, etc.) for PrEP indications. PrEP indications included one or more positive laboratory test results for chlamydia, gonorrhea, or active syphilis. Active syphilis was defined as a rapid plasma reagin (RPR) test ≥ 1:8. We also included anyone with injection drug use (IDU) noted in their social history. To mirror CDC clinical practice guidelines for PrEP, we created time periods to indicate when there was substantial risk of acquiring HIV infection [6]. Medical encounters that occurred during these time periods were examined for missed opportunities for PrEP initiation. For patients with positive STI results, we examined encounters for six months after the test order date. For IDU, we assumed continuous PrEP indication until documentation of cessation.

We removed medical encounters that we deemed unlikely to provide opportunities to prescribe PrEP. These encounters included medical procedures (imaging, dialysis, and intravenous treatments) and encounters with non-prescribing providers (physical therapy/rehabilitation, audiology, speech pathology, behavioral health, and nutrition). We categorized medical encounters to examine healthcare settings where PrEP was or was not prescribed. Healthcare settings included inpatient, emergency department (ED), primary care (including family medicine, primary care, urgent care settings, general pediatrics, and gerontology), infectious disease (ID), obstetrics and gynecology/women’s health (OBGYN/women’s health) and other outpatient settings (outpatient visits excluding OBGYN/women’s health, ID, or primary care).

PrEP prescriptions included prescription for combination therapy with tenofovir disoproxil fumarate/emtricitabine, tenofovir alafenamide/emtricitabine, or the ingredients prescribed concurrently (emtricitabine and tenofovir disoproxil fumarate or tenofovir alafenamide) without the addition of other antiretroviral therapy (ART). If other ART prescriptions were present, we assumed that the patient was on either post-exposure prophylaxis (PEP) or treatment for HIV and did not include these persons among those on PrEP. To assign PrEP prescriptions to specific medical encounters and healthcare settings, we included all encounters and healthcare settings two weeks prior to the order date of the PrEP prescription.

As data were obtained from two different healthcare institutions, research team consensus and standardization were sought before data combination and creation of variables. Demographic information included race, ethnicity, age, and sex as reported in the electronic medical record (EMR). Age was categorized as follows: under 18 years, 18–24 years, 25–34 years, 35–44 years, 45–54 years, and 55 years and older. Race and ethnicity information were combined to create the following categories: non-Hispanic Black (NHB), non-Hispanic white (NHW), Hispanic/Latino, Asian/Mideast Indian, more than one race, other, and unknown. To describe sexual behavior, we created categories using the sex of the patient in combination with the sex of their partner(s) and information on if the patient was currently sexually active. These categories included sexually active women who had sex with men (WSM), sexually active men who had sex with women (MSW), and sexually active men who had sex with men (MSM). To determine if prescribing patterns changed over time, we also examined encounters by year.

Descriptive statistics were performed using frequencies and percentages or medians and interquartile ranges (IQRs). Differences in characteristics were compared using chi-square tests for categorical variables or Wilcoxon rank-sum test for continuous variables. Bivariable and multivariable mixed effects logistic regression models were created to examine associations, reporting odds ratios or adjusted odds ratios and 95% confidence intervals (OR, aOR, 95% CI). Mixed effect models were used to allow for multiple encounters by the same patient, as well as changes in behavior over time for IDU, sexual activity, and sexual behavior. Two adjusted models were created to accommodate collinearity between documented sex and sexual behavior. All analyses included a variable to control or describe differences between the two institutions (institution A or B) while allowing for data from both institutions to be combined to provide a larger overview of the PrEP landscape in Chicago. For all models, complete case analysis was used, and if there were categories that did not vary by outcome (i.e., no PrEP prescriptions), this category was dropped from the model (i.e., as occurred when modeling missing age and missing sex). Data analyses were done in SAS (version 9.4, Cary, North Carolina) and R (version 4.3.0; R Core Team). This study was approved by the Institutional Review Boards at the University of Chicago (IRB22-0237) and Northwestern University (STU00202938).

## Results

In total, 9,664 people had one or more negative HIV tests as well as one or more PrEP indications during the seven-year study period. These participants contributed 53,031 medical encounters to this analysis. The number of people with PrEP indications was nearly equal at both institutions, with 46.6% from institution A and 53.4% from institution B. The two healthcare institutions had different patient demographic distributions, with A having a larger proportion of 18–24-year-olds (58.3% vs. 31.3%), NHB (83.8% vs. 27.9%), and women (65.7% vs. 46.3%). Institution A also had a higher proportion of women reporting sexual activity with men (33.9% vs. 23.0%) and patients with gonorrhea diagnoses (35.8% vs. 27.2%). Institution B had a greater proportion of NHW (40.6% vs. 7.1%), Hispanic/Latino patients (14.0% vs. 4.2%), men (50.5% vs. 34.3%), and men reporting sexual activity both with men and with women (MSM: 16.7% vs. 3.9%, MSW: 25.5% vs. 14.9%). Institution B also had a larger proportion of IDU (2.7% vs. 0.6%), and positive laboratory results indicating active syphilis infection (11.1% vs. 8.9%). Institutions A and B had similar proportions of people with positive chlamydia test results (72.5% vs. 72.8%) (Table 1).

**Table 1:** Characteristics of patients without HIV and indications for PrEP from 2015–2021 at two institutions in Chicago (*n* = 9,644)

	Total Number and percent (%) *N* = 9,664	Institution A Number and percent (%) *N* = 4,502	Institution B Number and percent (%) *N* = 5,162	*P*-value
Age (years)^α^ 18–24 25–34 35–44 45–54 55+ Missing	4,242 (43.89%) 3,491 (36.12%) 1,056 (10.93%) 435 (4.50%) 278 (2.88%) 162 (1.68%)	2,625 (58.31%) 1,382 (30.70%) 295 (6.55%) 95 (2.11%) 105 (2.33%) 0 (0.00%)	1,617 (31.33%) 2,109 (40.86%) 761 (14.74%) 340 (6.59%) 173 (3.35%) 162 (3.14%)	< 0.001
Race/ethnicity Non-Hispanic White Non-Hispanic Black Hispanic or Latino Non-Hispanic multiracial Non-Hispanic other Non-Hispanic unknown	2,416 (25.00%) 5,213 (53.94%) 912 (9.44%) 44 (0.46%) 322 (3.33%) 757 (7.83%)	321 (7.13%) 3,771 (83.76%) 187 (4.15%) 44 (0.98%) 102 (2.27%) 77 (1.71%)	2,095 (40.59%) 1,442 (27.93%) 725 (14.04%) 0 (0.00%) 220 (4.26%) 680 (13.17%)	< 0.001
Sex* Female Male Unknown	5,346 (55.32%) 4,155 (42.99%) 163 (1.69%)	2,956 (65.66%) 1,546 (34.34%) 0 (0.00%)	2,390 (46.30%) 2,609 (50.54%) 163 (3.16%)	< 0.001
Sexually active MSM^β^ No Yes	8,625 (89.25%) 1,039 (10.75%)	4,327 (96.11%) 175 (3.89%)	4,298 (83.26%) 864 (16.74%)	< 0.001
Sexually active WSM^β^ No Yes	7,338 (71.97%) 2,709 (28.03%)	2,978 (66.15%) 1,524 (33.85%)	3,977 (77.04%) 1,185 (22.96%)	< 0.001
Sexually active MSW^β^ No Yes	7,678 (79.45%) 1,986 (20.55%)	3,832 (85.12%) 670 (14.88%)	3,846 (74.51%) 1,316 (25.49%)	< 0.001
Indications for PrEP^β^				
Injection drug use	165 (1.71%)	28 (0.62%)	137 (2.65%)	< 0.001
Active syphilis infection	974 (10.08%)	399 (8.86%)	575 (11.14%)	< 0.001
Gonorrhea positive	3,013 (31.18%)	1,611(35.78%)	1,402 (27.16%)	< 0.001
Chlamydia positive	6,999 (72.42%)	3,262 (72.46%)	3,760 (72.84%)	0.005
PrEP Rx^β^ No Yes	8,723 (90.26%) 941 (9.74%)	4,399 (97.71%) 103 (2.29%)	4,324 (83.77%) 838 (16.23%)	< 0.001
Median number of encounters per person	3.00 [1.00, 6.00]	2.00 [1.00, 4.00]	4.00 [2.00, 9.00]	< 0.001

MSM = Men who have sex with men; WSM = Women who have sex with men; MSW = Men who have sex with women

*Documented sex

^α^Age at first encounter

^β^Ever reported, indicated or prescribed during the study time period

**Table 2:** Characteristics of encounters for patients without HIV and indications for PrEP from 2015–2021 at two institutions in Chicago (*n* = 53,031)

	Total Number and percent (%) *N* = 53,031	Institution A Number and percent (%) *N* = 16,270	Institution B Number and percent (%) *N* = 36,761	*P*-value
Site of care Emergency department Infectious disease Inpatient OBGYN/ women’s health Other Outpatient Primary Care	7,675 (14.47%) 1,158 (2.18%) 1,107 (2.09%) 13,540 (25.53%) 17,471 (32.94%) 12,080 (22.78%)	5,008 (30.78%) 514 (3.16%) 680 (4.18%) 5,518 (33.92%) 3,035 (18.65%) 1,515 (9.31%)	2,667 (7.25%) 644 (1.75%) 427 (1.16%) 8,022 (21.82%) 14,436 (39.27%) 10,565 (28.74%)	< 0.001
Sexually active MSM No Yes	43,989 (82.95%) 9042 (17.05%)	15,660 (96.25%) 610 (3.75%)	28,329 (77.06) 8432 (22.94)	< 0.001
Sexually active WSM No Yes	37,539 (70.79%) 15,492 (29.21%)	9477 (58.25%) 6793 (41.75%)	28,062 (76.34) 8699 (23.66)	< 0.001
Sexually active MSW No Yes	40,157 (75.72%) 12,874 (24.28%)	14,234 (87.49%) 2036 (12.51%)	25,923 (70.52%) 10,838 (29.48%)	< 0.001
Indications for PrEP				
Intravenous drug use	2362 (4.45%)	379 (2.33%)	1983 (5.39%)	< 0.001
Active syphilis infection	6171 (11.64%)	1646 (10.12%)	4525 (12.31%)	< 0.001
Gonorrhea positive	14,559 (27.45%)	4608 (28.32%)	9951 (27.07%)	0.003
Chlamydia positive	34,539 (65.13%)	11,419 (70.18%)	23,120 (62.89%)	< 0.001
PrEP Rx No Yes	48,378 (91.23%) 4653 (8.77%)	16,131 (99.15%) 139 (0.85%)	32,247 (87.72%) 4514 (12.28%)	< 0.001

MSM = Men who have sex with men; WSM = Women who have sex with men; MSW = Men who have sex with women

Most encounters took place at institution B (69.3%), which is reflected in the median visit count for patients being significantly higher at institution B as compared to institution A (median 4.00 IQR [2.00–9.00] vs. median 2.00 IQR [1.00–4.00], *p* < 0.001) (Tables 1 and 2). The most frequent settings for total eligible encounters were other outpatient settings (32.9%), followed by OBGYN/women’s health (25.5%), primary care (22.8%), and the ED (14.5%). Institution A contributed more ED encounters (65.3%) as well as inpatient encounters (61.4%) (Table 2). Institution B contributed more encounters to other outpatient setting (82.6%), ID (55.6%), OBGYN/women’s health (59.2%), and primary care (87.5%) (Table 2).

Most encounters were deemed opportunities for PrEP initiation due to being six months after a positive laboratory result for chlamydia (65.1%), followed by encounters six months after a positive laboratory result for gonorrhea (27.5%), followed by encounters six months after a positive laboratory result indicating active syphilis infection (11.6%), and finally due to IDU (4.5%). Overall, 941 patients received PrEP prescriptions, with the majority attending institution B (89.1%). These prescriptions were within a two-week window of 4,653 encounters (8.8% of all encounters).

When examining unadjusted associations between opportunities for PrEP and various characteristics, many factors were significantly associated with a PrEP prescription (Table 3). Attending institution B resulted in nearly six times the odds of being prescribed PrEP (OR 5.75 95% CI [3.39, 9.76]) than attending institution A. Being NHB, Hispanic/Latino, or of unknown race/ethnicity resulted in lower odds of PrEP prescription than being NHW, with odds ranging from being 0.08 times as likely to being 0.47 times as likely to obtain a PrEP prescription (NHB: OR 0.08 95% CI [0.05, 0.213], Hispanic/Latino: OR 0.36 95% CI (0.21, 0.64), and unknown race/ethnicity OR 0.47 95% CI [0.26, 0.86]). Being older than 24 years of age was associated with increased odds of PrEP prescription compared to individuals aged 18–24, ranging from 3.23 to 5.20 times as likely (25–34 years: OR 3.23 95% CI [2.24, 4.66], 35–44 years: OR 5.20 95% CI [3.43, 7.88], 45–54 years: OR 4.53 95% CI [2.73, 7.52], and 55+ years: OR 3.53 95% CI [1.84, 6.80]). Men had nearly 66 times the odds of obtaining a PrEP prescription as women (OR 63.16 95% CI [34.32, 116.23]). Having appointments in the ID department, primary care, or other outpatient setting all increased the odds of obtaining a PrEP prescription as compared to an encounter in the ED (ID: OR 11.43 95% CI [7.01, 18.64], other outpatient: OR 3.86 95% CI [2.56, 5.81], primary care: OR 4.05 95% CI [2.69, 6.12]). However, having an encounter in the OBGYN/women’s health department was associated with decreased odds of PrEP prescription (OR 0.04 95% CI [0.01, 0.24]). In terms of PrEP indications, only a recent positive laboratory result for chlamydia was significantly associated with PrEP prescription (OR 1.15 95% CI [1.01, 1.32]). Being a man who had sexual partners with either men or women was associated with increased odds of PrEP prescription compared to female patients or men not reporting these behaviors (MSM: OR 9.62 95% CI [6.76, 13.71], MSW: OR 4.44 95% CI [3.36, 5.88]). However, women who had sex with men had lower odds of PrEP prescription compared to male patients or women not reporting these behaviors (OR 0.03 95% CI [0.01, 0.10]). Having encounters in later years of the study period (2018 to 2021) was associated with greater odds of PrEP prescription, with more than twice the odds of that seen in 2015.

**Table 3:** Odds ratios and confidence intervals for mixed effects models examining PrEP prescriptions at the encounter level for a model containing sexual behavior (*n* = 53,031)

Variable	Unadjusted Odds Ratio 95% Confidence interval (lower, upper CI)	Adjusted Odds Ratio 95% Confidence interval (lower upper CI)
Institution A B	Reference 5.75 (3.39, 9.76)*	Reference 1.72 (1.19, 2.48)*
Race/ethnicity Non-Hispanic White Non-Hispanic Black Hispanic or Latino Non-Hispanic multiracial Non-Hispanic other Non-Hispanic unknown	Reference 0.08 (0.05, 0.13)* 0.36 (0.21, 0.64)* 0.14 (0.00, 5.99) 0.54 (0.23, 1.27) 0.47 (0.26, 0.86)*	Reference 0.21 (0.15, 0.29)* 0.53 (0.37, 0.76)* 0.32 (0.03, 3.32) 0.74 (0.44, 1.26) 0.80 (0.55, 1.18)
Age (*n* = 51,991) 18–24 25–34 35–44 45–54 55+	Reference 3.23 (2.24, 4.66)* 5.20 (3.43, 7.88)* 4.53 (2.73, 7.52)* 3.53 (1.84, 6.80)*	Reference 2.33 (1.75, 3.09)* 3.60 (2.61, 4.97)* 2.76 (1.87, 4.06)* 1.46 (0.87, 2.46)
Site of care Emergency department Infectious disease Inpatient OBGYN/Women’s Health Other Outpatient Primary Care	Reference 11.43 (7.01, 18.64)* 1.17 (0.47, 2.95) 0.04 (0.01, 0.24)* 3.86 (2.56, 5.81)* 4.05 (2.69, 6.12)*	Reference 10.27 (6.66, 15.83)* 0.93 (0.41, 2.13) 0.14 (0.06, 0.31)* 2.25 (1.57, 3.23)* 1.39 (0.85, 2.26)
Injection drug use No Yes	Reference 0.02 (0.00, 1.99)	Reference 0.01 (0.001, 0.08)*
Active syphilis infection^α^ No active syphilis infection Active syphilis infection	Reference 0.87 (0.71, 1.06)	Reference 0.99 (0.80, 1.23)
Chlamydia Positive Not chlamydia positive Chlamydia positive	Reference 1.15 (1.01, 1.32)*	Reference 1.03 (0.87, 1.21)
Gonorrhea positive Not gonorrhea positive Gonorrhea positive	Reference 0.90 (0.78, 1.03)	Reference 0.99 (0.84, 1.16)
Sexually active MSM No Yes	Reference 9.62 (6.76, 13.71)*	Reference 22.63 (14.47, 35.38)*
Sexually active WSM No Yes	Reference 0.03 (0.01, 0.10)*	Reference 0.10 (0.06, 0.17)*
Sexually active MSW No Yes	Reference 4.44 (3.36, 5.88)*	Reference 0.37 (0.24, 0.57)*
Year of patient encounter 2015 2016 2017 2018 2019 2020 2021	Reference 0.91 (0.50, 1.66) 0.79 (0.45, 1.40) 2.03 (1.28, 3.22)* 2.52 (1.60, 3.97)* 2.06 (1.30, 3.27)* 2.15 (1.35, 3.41)*	Reference 0.66 (0.39, 1.11) 0.47 (0.28, 0.78)* 1.47 (0.97, 2.20) 1.83 (1.22, 2.73)* 1.46 (0.97, 2.20) 1.40 (0.93, 2.11)

MSM = Men who have sex with men; WSM = Women who have sex with men; MSW = Men who have sex with women

*Significant finding (*p* < 0.05)

^α^Active syphilis infection was indicated by RPR ≥ 1:8

When examining adjusted associations in a model including sexual behavior but not sex, results were similar to those seen in unadjusted analyses, although generally the magnitude of point estimates and confidence intervals were attenuated (Table 3). A few results were no longer significant, including the odds of PrEP prescription for those aged 55 years and older (aOR 1.46 95% CI [0.87, 2.46]) and those who received a recent positive test result for chlamydia (aOR 1.03 95% CI [0.87, 1.21]). However, the odds PrEP prescription for sexually active MSM increased from 9.62 (95% CI [6.76, 13.71]) to 22.63 (95% CI [14.47, 35.38]). The odds of PrEP prescription for people with IDU also became more extreme, with even lower odds (aOR 0.01 95% CI [0.001, 0.08]). The association for MSW in the sexual behavior adjusted model reversed direction, from 4.44 times to 0.37 times the odds of PrEP prescription (95% CI [0.24, 0.57]) (Table 3). For year of encounter, 2017 gained significance in adjusted models, showing lower odds of PrEP prescription compared to 2015 (aOR 0.47 95% CI [0.28, 0.78]). Conversely, year of encounter lost significance for 2018 (aOR 1.47 95% CI [0.97, 2.20]), 2020 (aOR 1.46 95% CI [0.97, 2.20]), and 2021 (aOR 1.40 95% CI [0.93, 2.11]).

When examining the adjusted model including sex, results were largely similar to those obtained when modeling sexual behavior (Supplemental Table). Adjusted models containing sex resulted in increased odds for men regardless of the sex of their partner, although these results were slightly attenuated from unadjusted results (aOR 42.56, 95% CI [27.67, 65.46]).

## Discussion

Underutilization of PrEP can only improve if we understand prescribing patterns and opportunities to offer PrEP among patients with clear indications. In this study, we examined PrEP prescribing at two large academic healthcare systems in Chicago and found opportunities to improve awareness of PrEP and PrEP delivery in settings where patients are accessing sexual health services, including the ED and OBGYN/women’s health clinics. In this analysis of patients who had documented indications for PrEP, only 10% received a prescription. This prescription rate is slightly higher than studies with similar settings that have found PrEP prescription rates between 1% and 8% [17–19].

We found that persons who were NHB or Hispanic/Latino, as well as younger patients, were less likely to be prescribed PrEP. These disparities persisted despite controlling for site, indicating that regardless of prescribing institution or the demographics of the institution’s patient population, some people were less likely to obtain a PrEP prescription. Indeed, age-related disparities have been reported in other studies as well, which have found an association between older age and increased odds of PrEP prescription [18, 20]. Moreover, data on PrEP prescriptions demonstrate that despite increases in PrEP use over the past decade, it varies greatly by race and ethnicity. In 2022, 64% of PrEP users were white, whereas 14% were Black and 17% were Hispanic/Latino. However, white people constituted 26% of new HIV diagnoses, while Black and Hispanic/Latino people accounted for 69% of new HIV diagnoses [21]. There is a clear gap in PrEP uptake among Black and Hispanic/Latino people despite their need.

In addition to racial-, ethnic-, and age-related disparities, we also found that males had significantly higher odds of receiving a PrEP prescription compared to females. In the adjusted models, this result was largely driven by prescriptions for MSM as opposed to MSW. Yumori et al. investigated missed opportunities for PrEP among individuals testing positive for an STI at a large academic medical center and found that men were more likely to have multisite STI testing, receive screening for syphilis and HIV, and be engaged in discussions about PrEP compared to women [22]. In another analysis of patients seen at an urban municipal medical center, despite women accounting for over half the patients with an indication for PrEP, none received a PrEP prescription [23]. In these studies, men typically have “high risk sexual behavior” as their primary indication for PrEP; however, the most common indication for PrEP among women is having a sexual partner living with HIV [17, 18, 24, 25]. Comprehensive documentation within the EMR of female sexual behaviors and risks other than having a partner with HIV is critical to adequately address this gap in HIV prevention [26].

It is difficult to determine if biases among healthcare providers are responsible for the disparities seen in PrEP prescriptions. While systemic bias is indicated by the similarity of our findings to that of previous work, little research has been done quantifying bias in PrEP prescriptions among providers. In work by Calabrese et al. that presented mock scenarios to identify biases related to PrEP clinical decision-making, little evidence was found for racism, although heterosexism by providers was indirectly associated with a lower intent to prescribe PrEP [27]. Later work that examined intent to prescribe among WSM and people with IDU found that providers’ concerns about PrEP adherence resulted in lower intent to prescribe for both groups compared to MSM [28]. Similar studies among pharmacy students have shown how a patient’s race can negatively influence prescriber confidence in providing PrEP-related care to Black patients [29].

In large healthcare systems with acute ED- and hospital-based care, as well as primary and subspecialty care, targeted efforts to increase PrEP awareness and uptake in areas where indicated patients are accessing care may be more effective than a systems-wide initiative. In this study, PrEP prescriptions were associated with encounters in ID, primary care, and other subspecialty settings, though many encounters for patients with PrEP indications also occurred in the ED and OBGYN/women’s health, where we found reduced odds of PrEP prescriptions. In the ED, collecting information on sexual behavior and STIs may facilitate identifying patients who might benefit from PrEP. In the OBGYN/women’s health setting, patients are overwhelmingly heterosexual women who are often not considered to be at risk for HIV. In Chicago, however, women account for approximately 30% of new HIV diagnoses, which is much higher than the national average [30, 31]. Furthermore, clinician confidence in discussing or initiating PrEP may be a barrier, particularly in the ED where more acute medical concerns are likely prioritized and workflows for PrEP prescribing and PrEP follow-up care may not be clearly established.

Given the importance of PrEP as a HIV prevention tool, it was hoped that in adjusted models PrEP prescriptions would show significant and continued increases each year. However, we did not see consistent increases in PrEP prescriptions by year of patient encounter. It is troubling that compared to the control year of 2015, there was only one year (2019) throughout the entire study period during which there was a significant increase in PrEP prescriptions. This indicates the need for widespread patient and provider education regarding the importance of PrEP at our institutions, as well as comprehensive implementation strategies to bridge this gap.

Our results can help guide interventions or health system policy changes, yet additional information on barriers to prescribing PrEP should be considered. Qualitative studies have identified barriers including insufficient provider training, competing priorities, limited time during appointments, patients’ concerns about potential side effects, and provider workload [32]. Important strategies for reducing barriers include provider training, protocols for PrEP prescribing, and clinical decision support tools such as EMR alerts for patients with PrEP indications. A randomized trial that evaluated an intervention that used an EMR-based HIV risk algorithm found no significant increase in PrEP initiation among all patients; however, among patients whose primary care providers also provided care for those with HIV, there was a significant increase in PrEP initiation [33]. This could suggest that clinical decision support may be effective in improving PrEP provision among providers who are already familiar with PrEP, whereas more intensive interventions may be required for PrEP naïve providers or those who are unfamiliar with HIV and HIV prevention. Additionally, use of EMR-based algorithms are dependent on accurate and thorough documentation of sexual behaviors in the medical record, which may not always be present. We found that these behaviors were likely to be under-documented in the EMR in structured fields from at least one participating institution. Other solutions, such as same-day sexual health appointments for those presenting with sexual health or STI-related concerns, may be beneficial, even for those presenting to the ED [15, 34–36]. Other authors have suggested methods to streamline PrEP prescribing such as PrEP navigation or same-day initiation, as well as easing the complexity of PrEP follow-up care that deters many providers from prescribing PrEP [35, 36].

Finally, it is difficult to determine the degree to which patient response to PrEP or demand for PrEP may be responsible for obtaining a PrEP prescription among those with indications. Patient opinions or lack of familiarity with PrEP may result in refusal of PrEP after providers offer it. A lack of perceived vulnerability to HIV may also influence acceptance of a PrEP prescription among those indicated. Studies have shown that people affected by IDU may consider themselves less likely to acquire HIV, despite risks that are posed by sharing injection equipment allowing for blood borne disease transmission [37]. Similarly, women may consider themselves at low risk for HIV, which might make them less likely to agree to receiving a prescription for PrEP [38, 39]. Finally, social drivers of health, such as lack of insurance coverage or payment options for PrEP, competing priorities, and IDU, may have impacted patients’ interest in PrEP and inadvertently contributed to the prescribing disparities seen in this analysis [40, 41].

This study should be considered in light of its limitations. Some information, including insurance data, was unavailable from both healthcare systems, and thus could not be included in the analysis. Social, sexual, and drug use behaviors may not have been consistently or accurately collected within structured fields in the EMR, which may have led to indications for PrEP being misclassified. Interpretation of syphilis test results, especially determination of active versus past infection, can be difficult. Our use of the RPR cutoff of *≥* 1:8 may have excluded some patients with active syphilis who would have been indicated for PrEP; thus, a more nuanced analysis of syphilis indicators for PrEP would be informative. Using all encounters within a two-week window of PrEP prescriptions may have overestimated the actual number and variety of settings that prescribed PrEP. However, our approach did consider timing from prescription order to patient pickup, as well as delays from insurance prescription drug coverage. Unfortunately, it is unknown how many patients declined PrEP when offered it or how many patients received education about PrEP but not a prescription. Lastly, although we wanted to include interaction terms in our adjusted models to examine if disparities in PrEP prescriptions lessened over time, models containing interaction terms failed to converge due to small PrEP prescriptions among some groups when subdivided by year. Strengths of our study include the use of data from two different healthcare systems across Chicago and the length of the study period.

## Conclusions

In this dual-institution study of PrEP prescribing, we found opportunities to improve PrEP prescribing where patients are accessing care for sexual health. We also identified opportunities to improve equitable PrEP prescribing among young Black and Latino populations, as well as women highly impacted by HIV. Our results can inform targeted interventions at the healthcare system level to improve PrEP prescribing and uptake for indicated patients.

## Electronic Supplementary Material

Below is the link to the electronic supplementary material.

Supplementary Material 1
