# Supplementary material for: Understanding Opportunities for Prescribing Pre-exposure Prophylaxis at Two Academic Medical Centers in a High Priority Jurisdiction for Ending the HIV Epidemic
Source: AIDS Behav. 2025 Jun 10;29(10):3162–71. doi: 10.1007/s10461-025-04767-y (PMC12484277; doi:10.1007/s10461-025-04767-y)
Supplement: Supplementary file 1 — Supplementary Material 1 [file 10461_2025_4767_MOESM1_ESM.docx]

**Supplemental Table: Odds ratios and confidence intervals for mixed effects models examining PrEP prescriptions at the encounter level for a model containing sex (n=53,031)**

| **Variable** | **Unadjusted Odds Ratio 95% Confidence interval (lower, upper CI)** | **Adjusted Odds Ratio 95% Confidence interval**  **(lower, upper CI)** |
| --- | --- | --- |
| **Institution**  A  B | Reference  5.75 (3.39, 9.76)* | Reference  2.01 (1.34, 3.02)* |
| **Race/ethnicity**  Non-Hispanic White  Non-Hispanic Black  Hispanic or Latino  Non-Hispanic multiracial  Non-Hispanic other  Non-Hispanic unknown | Reference  0.08 (0.05, 0.13)*  0.36 (0.21, 0.64)*  0.14 (0.00, 5.99)  0.54 (0.23, 1.27)  0.47 (0.26, 0.86)* | Reference  0.14 (0.10, 0.21)*  0.52 (0.34, 0.78)*  0.23 (0.02, 2.89)  0.64 (0.35, 1.17)  0.60 (0.38, 0.93)* |
| **Age (n=51,991)**  18-24  25-34  35-44  45-54  55+ | Reference  3.23 (2.24, 4.66)*  5.20 (3.43, 7.88)*  4.53 (2.73, 7.52)*  3.53 (1.84, 6.80)* | Reference  2.25 (1.65, 3.07)*  3.39 (2.38, 4.82)*  2.33 (1.53, 3.56)*  1.23 (0.70, 2.17) |
| **Sex**^α^ **(n=51,973)**  Female  Male | Reference  63.16 (34.32, 116.23)* | Reference  42.56 (27.67, 65.46)* |
| **Site of care**  Emergency department  Infectious disease  Inpatient  OBGYN/ women's health  Other Outpatient  Primary Care | Reference  11.43 (7.01, 18.64)*  1.17 (0.47, 2.95)  0.04 (0.01, 0.24)*  3.86 (2.56, 5.81)*  4.05 (2.69, 6.12)* | Reference  9.32 (5.95, 1.46)*  1.18 (0.50, 2.79)  0.51 (0.20, 1.27)  2.18 (1.49, 3.19)*  1.55 (0.92, 2.59) |
| **Injection drug use**  No  Yes | Reference  0.02 (0.00, 1.99) | Reference  0.00 (0.00, 0.04)* |
| **Active syphilis infection**^β^  No active syphilis infection  Active syphilis infection | Reference  0.87 (0.71, 1.06) | Reference  0.97 (0.78, 1.21) |
| **Chlamydia Positive**  Not chlamydia positive  Chlamydia positive | Reference  1.15 (1.01, 1.32)* | Reference  1.02 (0.86, 1.21) |
| **Gonorrhea positive**  Not gonorrhea positive  Gonorrhea positive | Reference  0.90 (0.78, 1.03) | Reference  0.99 (0.83, 1.17) |
| **Year of patient encounter**  2015  2016  2017  2018  2019  2020  2021 | Reference  0.91 (0.50, 1.66)  0.79 (0.45, 1.40)  2.03 (1.28, 3.22)*  2.52 (1.60, 3.97)*  2.06 (1.30, 3.27)*  2.15 (1.35, 3.41)* | Reference  1.02 (0.60, 1.74)  0.86 (0.51, 1.44)  2.88 (1.90, 4.37)*  3.69 (2.44, 5.57)*  3.00 (1.98, 4.55)*  3.01 (1.98, 4.58)* |

MSM = Men who have sex with men; WSM = Women who have sex with men; MSW = Men who have sex with women

*Significant finding (p<0.05)

^α^Documented sex

^β^Active syphilis infection was indicated by RPR ≥ 1:8
